# FTO-dependent m^6^A regulates muscle fiber remodeling in an NFATC1–YTHDF2 dependent manner

**DOI:** 10.1186/s13148-023-01526-5

**Published:** 2023-07-05

**Authors:** Wengang Wang, Xueming Du, Ming Luo, Ningning Yang

**Affiliations:** 1 Department of Orthopedics, The First Affiliated Hospital of Zhengzhou University, Zhengzhou, 450052 People’s Republic of China; 2 Department of Gynaecology and Obstetrics, The Third Affiliated Hospital of Zhengzhou University, Zhengzhou, 450052 Henan Province People’s Republic of China; 3 Department of Orthopedics, Zhongnan Hospital of Wuhan University, Wuhan, 430071 People’s Republic of China; 4 Department of Emergency, The First Affiliated Hospital of Zhengzhou University, Zhengzhou, 450052 People’s Republic of China

**Keywords:** FTO, m^6^A demethylation, Muscle fiber remodeling, Adolescent idiopathic scoliosis

## Abstract

**Background:**

Adolescent idiopathic scoliosis (AIS) is characterized by low lean mass without vertebral deformity. The cause-and-effect relationship between scoliosis and paraspinal muscle imbalance has long puzzled researchers. Although *FTO* has been identified as a susceptibility gene for AIS, its potential role in the asymmetry of paraspinal muscles has not been fully elucidated.

**Methods:**

We investigated the role of *Fto* in murine myoblast proliferation, migration, and myogenic differentiation. We examined its precise regulatory influence on murine muscle fiber remodeling in vitro and in vivo. We identified the downstream target gene of *Fto* by screening key regulators of murine muscle fiber remodeling and identified its m^6^A reader. Deep paraspinal muscle samples were obtained from the concave and convex sides of AIS patients with or without Schroth exercises, and congenital scoliosis served as a control group. We compared the content of type I fibers, expression patterns of fast- and slow-type genes, and levels of FTO expression.

**Results:**

FTO contributed to maintain the formation of murine slow-twitch fibers both in vitro and in vivo. These effects were mediated by the demethylation activity of FTO, which specifically demethylated NFATC1 and prevented YTHDF2 from degrading it. We found a significant reduction in type I fibers, mRNA levels of *MYH7* and *MYH7B*, and expression of FTO on the concave side of AIS. The percentage of type I fibers showed a positive correlation with the expression level of *FTO*. The asymmetric patterns observed in AIS were consistent with those seen in congenital scoliosis, and the asymmetry of FTO expression and fiber type in AIS was largely restored by Schroth exercises.

**Conclusions:**

FTO supports the formation of murine slow-twitch fibers in an NFATC1–YTHDF2 dependent manner. The consistent paraspinal muscle features seen in AIS and congenital scoliosis, as well as the reversible pattern of muscle fibers and expression of FTO in AIS suggest that *FTO* may contribute to the muscle fiber remodeling secondary to scoliosis.

**Supplementary Information:**

The online version contains supplementary material available at 10.1186/s13148-023-01526-5.

## Introduction

Adolescent idiopathic scoliosis (AIS) is one of the most common spinal deformities, affecting millions of children with an incidence of about 3–4% worldwide [1]. A curve magnitude greater than 45 degrees is the surgical threshold for treating AIS [2]. Surgical treatment may induce trauma and psychological effects, such as neurovascular injury, internal fixation failure, and restriction of back movement [3]. Unlike congenital scoliosis (CS), which is characterized by hemivertebral deformity, AIS is distinguished by low lean mass without vertebral deformities. Additionally, low lean mass of the back muscles is associated with a higher risk of curve progression in AIS [4]. Although several genes have been associated with AIS, their specific roles in the development of the condition remain unclear. Recent research has given increasing attention to the role of epigenetics in the development of AIS [5–7]. While *FTO* has been identified as a susceptibility locus for AIS based on a genome-wide association analysis involving 79,211 people, its specific role in the etiology of AIS remains unclear [8].

AIS is believed to be caused by an imbalance of the paraspinal muscles, with the concave paraspinal muscles having a lower percentage of type I fibers than the convex [9]. The slow-twitch and fast-twitch fiber types of skeletal muscle are different from one another in terms of contractile protein composition, oxidative capacity, and preferred substrate for ATP synthesis [10]. Muscle fiber-type switching depends on the exact coordination of metabolic and contractile gene expression programs to control fiber-type specification and ensure contractile function [11]. FTO is well-known for its connection to obesity and its crucial role in controlling transcriptome-wide m^6^A modification [12]. Previous studies have demonstrated that *FTO* is necessary for myogenesis [13, 14]. However, the relevance of FTO-dependent m^6^A demethylation in fiber type-specific patterns and its role in skeletal muscle fiber remodeling have not been discussed.

To investigate the role of the *FTO* susceptibility gene in AIS and its potential function in skeletal muscle fiber remodeling via the FTO-dependent m^6^A epitranscriptome, we used paraspinal muscle samples from AIS patients, a mouse model of muscle injury, and murine myoblast culture methods. We found that FTO promotes the development of murine slow-twitch fibers both in vitro and in vivo, primarily through its demethylation of NFATC1 and prevention of YTHDF2-mediated degradation. Additionally, we demonstrated that the asymmetry in muscle fibers and FTO expression was not limited to AIS and could be reversed.

## Materials and methods

### Human samples

The current study adhered to the ethical guidelines outlined in the 1964 Declaration of Helsinki and its subsequent amendments after receiving approval from the Institutional Review Board. Participants in the study were scoliosis patients who met the following inclusion and exclusion criteria: (1) confirmed diagnosis of either AIS or CS and scheduled for one-stage posterior spinal surgery; (2) primary right thoracic curve; (3) female; (4) aged between 12 and 18 years; (5) Han ethnicity; (6) informed consent from guardians. Exclusion criteria included: (1) spinal deformities other than AIS or CS, such as neuromuscular scoliosis; (2) history of spinal surgery; (3) mental or psychological conditions preventing compliance with treatment.

The study enrolled twenty AIS and five CS patients who underwent posterior spinal surgery, as well as five additional AIS patients who had undergone Schroth exercises for more than three months. Deep paraspinal muscle biopsies were taken from the concave and convex sides of the curve apex during spinal surgery, and these samples were subsequently mounted for fiber typing or flash-frozen in liquid nitrogen for RNA and protein extraction. To minimize any confounding factors from individual variability that may impact our analysis results, we utilized a strict paired research design in our case selection. Although the sample size for the three groups (AIS, CS, and AIS with Schroth exercises) used for subsequent immunofluorescence and Western blot analyses was small, consisting of only five individuals each, all participants were female with a right thoracic curve and exhibited no significant differences in age or primary Cobb angle. Patient characteristics are provided in Additional file 1: Table S1.

### Animal samples

We used 6-week-old C57/BL mice to study the role of *Fto* in muscle fiber remodeling in vivo after receiving ethics committee approval. In brief, 50 µl of cardiotoxin (CTX, 10 μM, Latoxan) was intramuscularly injected into the soleus to induce acute muscle damage. To prevent the impact of *Fto* on the proliferation of satellite cells, we utilized FB23-2 (a specific inhibitor of FTO m^6^A demethylase) five days after CTX injection. 50 µl of FB23-2 (5 μM, AbMole) was injected into the soleus every three days. After five rounds of FB23-2 injections, we collected soleus biopsies for further immunofluorescence and quantitative real-time PCR analysis.

### Cell culture, transfection and tracking

C2C12 cells were cultured at 37 °C with 5% CO_2_ in growth media (DMEM containing 10% fetal bovine serum and 1% penicillin/streptomycin). To induce myogenic differentiation, C2C12 cells were grown to approximately 80% confluence and then switched to a differentiation medium (DMEM containing 2% horse serum and 1% penicillin/streptomycin). All cellular experiments were performed with biological replicates in 3–5 independent wells. Three independent siRNAs for *Fto* or *Ythdf2* were transfected into cells using Lipofectamine™ RNAiMAX Reagent (Invitrogen) according to the manufacturer’s instructions. The siRNAs were designed and synthesized by RIBOBIO. The target sequences of siRNAs are provided in Additional file 2: Table S2.

Live cell tracking was performed according to the manufacturer’s guidelines of Harmony High-Content Imaging and Analysis Software (PerkinElmer). Briefly, after 24 h of cell seeding, cells were placed into the high-content imaging system with conditions set at 37℃ and 5% CO_2_. Under bright field conditions, the software could identify all cells within the visual range. Cell recording was conducted at 15-min intervals with a total tracking time of 6 h. By continuously capturing cellular motion for 6 h and using the software to segment cells, various indicators of cell migration, including the accumulated distance, displacement per track, and average speed per track, were calculated. Objects detected at only one time point were treated as detection errors, and the software automatically corrected them through splitting or merging procedures.

### Immunofluorescence analysis

Paraspinal muscle biopsies from scoliosis patients and mice were frozen in precooled isopentane for immunofluorescence staining, and the frozen tissues were sectioned into 8 µm thick sections. After fixation with 4% paraformaldehyde and blocked with 5% BSA (Biofroxx), the sections were incubated with primary antibodies against MHC1 (1:20, DSHB) overnight at 4 °C. The sections were then incubated with secondary antibodies (Alexa Fluor 488 Anti-Mouse IgG or Alexa Fluor 594 Anti-Mouse IgG; 1:200, Yeasen) at room temperature for 2 h in the dark.

C2C12 cells and differentiated myotubes were fixed with 4% paraformaldehyde and then treated with 0.5% Triton® X-100 (Biofroxx). After blocking, cells were stained with primary antibodies against Ki67 (1:200, Proteintech), FTO (1:200, Proteintech), MHC1 (1:20, DSHB), and MyoG (1:100, Santa Cruz). Cells were then incubated with secondary antibodies (Alexa Fluor 488 Anti-Mouse IgG, Alexa Fluor 594 Anti-Rabbit IgG, or Alexa Fluor 594 Anti-Mouse IgG; 1:200, Yeasen) at room temperature for 2 h. Nuclei were stained with DAPI for 5 min.

The fluorescence microscope used for imaging was an ECLIPSE Ti2 (Nikon), and the positive signal threshold was determined by staining with negative control IgG. Five fields were randomly selected for each independent sample, and the sample sizes for each experimental group were specified in the figure legends. Image quantification was performed using ImageJ software (Version 1.52 V). The proportion of type I fibers was calculated as the percentage of type I fibers among the total muscle fibers in the muscle tissue cross-section. The fusion index of type I fibers was calculated by dividing the number of nuclei in the MHC1-positive region by the total number of nuclei in the field of view. The Ki67 and MyoG positivity rates were calculated as the percentage of positively stained cells in the field of view out of the total number of cells in the field of view, respectively. Individual data of immunofluorescence analysis were derived from three repeated measurements.

### Quantitative real-time PCR and Western blot assay

After the muscle samples were pulverized using a mortar and pestle in liquid nitrogen or the cells were washed with PBS, RNAiso (Takara) was added, and total RNA was isolated with Qiagen RNAeasy Mini Kits (Qiagen) according to the manufacturer’s guidelines. cDNA was synthesized using the PrimeScript™ RT reagent kit (Takara). In brief, 1 μg total RNA was mixed with 1 μl of gDNA Eraser and RNase free dH_2_O was added to a final volume of 10 μl for genomic DNA removal. The mixture was incubated at room temperature for 5 min. Additionally, a 10 μl Master Mix was prepared, comprising 1 μl PrimeScript RT Enzyme Mix I, 1 μl RT Primer Mix, 4 μl 5 × PrimeScript Buffer 2, and 4 μl RNase free dH_2_O. The Master Mix was then mixed with the total RNA. Amplification was carried out in a SimpliAmp™ Thermal Cycler (Thermo Fisher Scientific) under the following amplification conditions: incubation at 37 °C for 15 min followed by incubation at 85 °C for 5 s. PowerUp™ SYBR™ green master mix (Thermo Fisher Scientific) was added to detect the expression of related genes. In brief, a 10 μl component was prepared for the PCR reactions, including 5 μl 2X PowerUp™ SYBR™ Green Master Mix, 0.5 μl forward primer, 0.5 μl reverse primer, and 4 μl DNA template with RNase free dH_2_O. Quantitative real-time PCR was performed using the standard reaction mode with an ABI QuantStudio 3 machine (Thermo Fisher Scientific), including UDG activation at 50 °C for 2 min, activation at 95 °C for 2 min, denature at 95 °C for 15 s, extend at 60 °C for 1 min. We used delta delta Ct method to quantitation, and the internal reference gene GAPDH was chosen as the normalization gene to standardize the expression levels of the target gene relative to the reference gene in the samples. The gene-specific primers are listed in Additional file 3: Table S3 and Additional file 4: Table S4.

The total protein was extracted using a RIPA lysis solution containing 1% PMSF. Protein quantification was achieved using the BCA Protein Assay Kit (Biosharp). The extracted protein was mixed with SDS PAGE protein sample buffer in a 1:1 volume ratio and heated in a water bath at 100 °C for 15 min to fully denature the protein. The sample volume for the loading of each lane was calculated based on the protein quantification result, with 20 μg of protein loaded per lane with a sample volume of 15 μl. Gel electrophoresis and membrane transfer were conducted in accordance with standard processes. After blocking with 5% BSA to prevent nonspecific antigen binding, the membranes were incubated overnight with primary antibodies including FTO (1:1000, Proteintech), NFATC1 (1:1000, Proteintech), and GAPDH (1:5000, Proteintech). The membranes were incubated at room temperature for 2 h following the secondary antibody incubation (HRP-conjugated goat anti-rabbit IgG secondary antibody; 1:10,000, Yeasen). After incubation, the membrane was washed 3 times with TBST buffer for 15 min each time. Signal detection was conducted using the Enhanced ECL chemiluminescence detection kit (Vazyme), and image acquisition was completed using the ChemiDocTM Imaging System. The gray value for the target protein was analyzed using ImageJ software (Version 1.52v), with individual data derived from three repeated measurements.

### Quantification of m^6^A modification and RNA stability assays

For quantification of m^6^A in human and mouse skeletal muscle tissue as well as in C2C12 cells during differentiation, an EpiQuik m^6^A RNA Methylation Quantification Kit (Epigentek) was used following the manufacturer’s protocol. The percentage of m^6^A in total RNA was calculated with a colorimetric quantification method by reading the absorbance at 450 nm.

C2C12 cells were transfected with *Fto* siRNAs when they reached approximately 60% confluence. After being cultured in complete medium for an additional 24 h, the cell density reached around 80%. Then, the growth medium was replaced with a differentiation medium for subsequent experiments. Cells were then treated with actinomycin D (5 μg/mL, AbMole) to inhibit global mRNA transcription, and cells were harvested at 0, 3, and 6 h to assess RNA stability. The expression levels of target genes were detected with quantitative real-time PCR.

### Statistical analysis

Data were analyzed and plotted using GraphPad Prism (Version 8.4.3). The data for individual samples was obtained by taking the average of three measurements. For samples in the same case series, we further process the quantified indicators of individual samples as the mean ± s.d. (e.g., In the case of a patient with AIS, we conducted three repeated measurements of their Cobb angle, resulting in an average Cobb angle of 53.4°. We then processed the Cobb angles of 5 AIS patients and summarized the data for this case series as 47.4° ± 3.9°). Non-parametric tests were performed due to the small sample size in each series. For the detection of para-vertebral muscle on the concave and convex sides of the same patient, paired t-tests were utilized, while independent samples were tested using non-paired *t*-tests. After passing the normality test, Pearson correlation analysis was used to explore the correlation between *FTO* mRNA expression levels and the proportion of type I fibers. One-way ANOVA was used to compare the data among the three groups. A *P* value of < 0.05 was considered statistically significant.

## Results

### *Fto* regulates the proliferation and migration of murine myoblasts

**Fig. 1:**
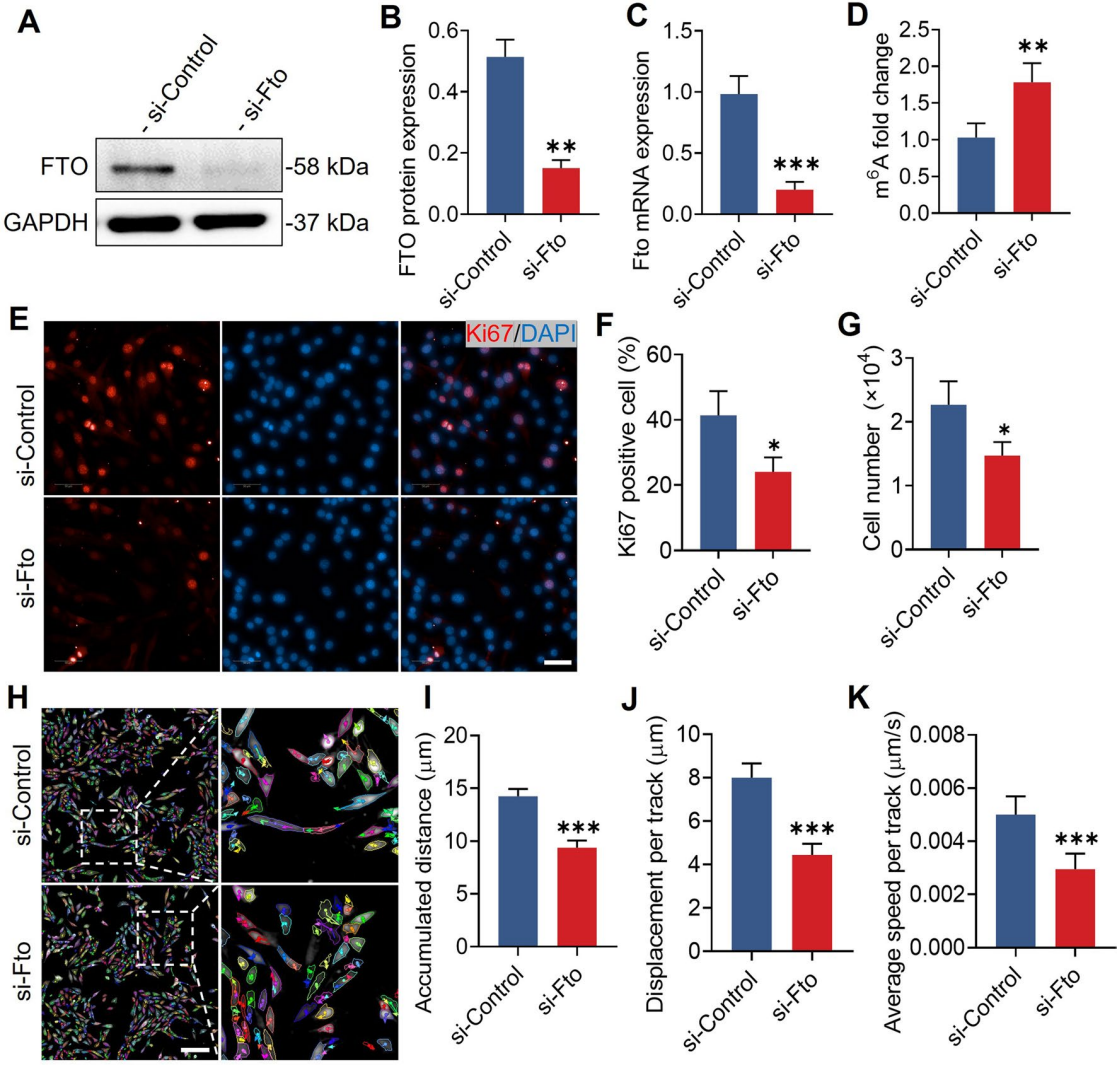
*Fto* regulates the proliferation and migration of murine myoblasts. **A** Representative Western blot analysis of si-*Fto* interference in C2C12 cells. **B** Western blot quantification verified the knock-down efficiency of si-*Fto* interference. n = 3 in each case; data are represented as mean ± s.d. **C** Quantitative real-time PCR analysis verified the knock-down efficiency of si-*Fto* interference. n = 5 in each case; data are represented as mean ± s.d. **D** The m^6^A level was significantly increased after si-*Fto* interference. n = 5 in each case; data are represented as mean ± s.d. **E** Immunofluorescence staining of Ki67 after si-*Fto* interference in myoblasts. Scale bar, 50 μm. **F** Interference with si-*Fto* decreased the percentage of Ki67-positive cells. n = 5 in each case; data are represented as mean ± s.d. **G** Cell count analysis showed consistent trends with immunofluorescence analysis of Ki67. n = 5 in each case; data are represented as mean ± s.d. **H** Representative images of the live cell tracking technique. Scale bar, 200 μm. **I** The accumulated distance was significantly decreased in si-*Fto*-treated myoblasts. n = 5 in each case; data are represented as mean ± s.d. **J** Displacement per track was significantly decreased in si-*Fto* treated myoblasts. n = 5 in each case; data are represented as mean ± s.d. **K** Average speed per track was significantly decreased in si-*Fto* treated myoblasts. n = 5 in each case; data are represented as mean ± s.d. *P < 0.05, **P < 0.01, ***P < 0.001

In order to investigate the potential role of *Fto* in murine myoblasts, we utilized siRNAs against *Fto* (si-*Fto*) in C2C12 cells to lose its function. The knockdown efficiency of si-*Fto* was verified with quantitative real-time PCR, and Western blot analysis (Fig. 1A–D). Murine myoblasts treated with si-*Fto* showed a considerably lower proportion of Ki67-positive cells (Fig. 1E–F). Similarly, we found that the total cell number of murine myoblasts was significantly decreased after si-*Fto* treatment (Fig. 1G). To assess the effect of *Fto* on murine myoblast migration, we used a live cell tracking technique to minimize the influence of *Fto*-regulated cell proliferation and low serum-induced myoblast differentiation. We cultured C2C12 cells in growth media and tracked live cells for 6 h using a high-content screening instrument (Fig. 1H). The accumulated distance, displacement per track, and average speed per track were significantly decreased in si-*Fto* treated myoblasts (Fig. 1I–K). In conclusion, these data indicate that *Fto* maintains the proliferation and migration of murine myoblasts.

### *Fto* maintains the expression of murine slow-twitch fiber related genes

**Fig. 2:**
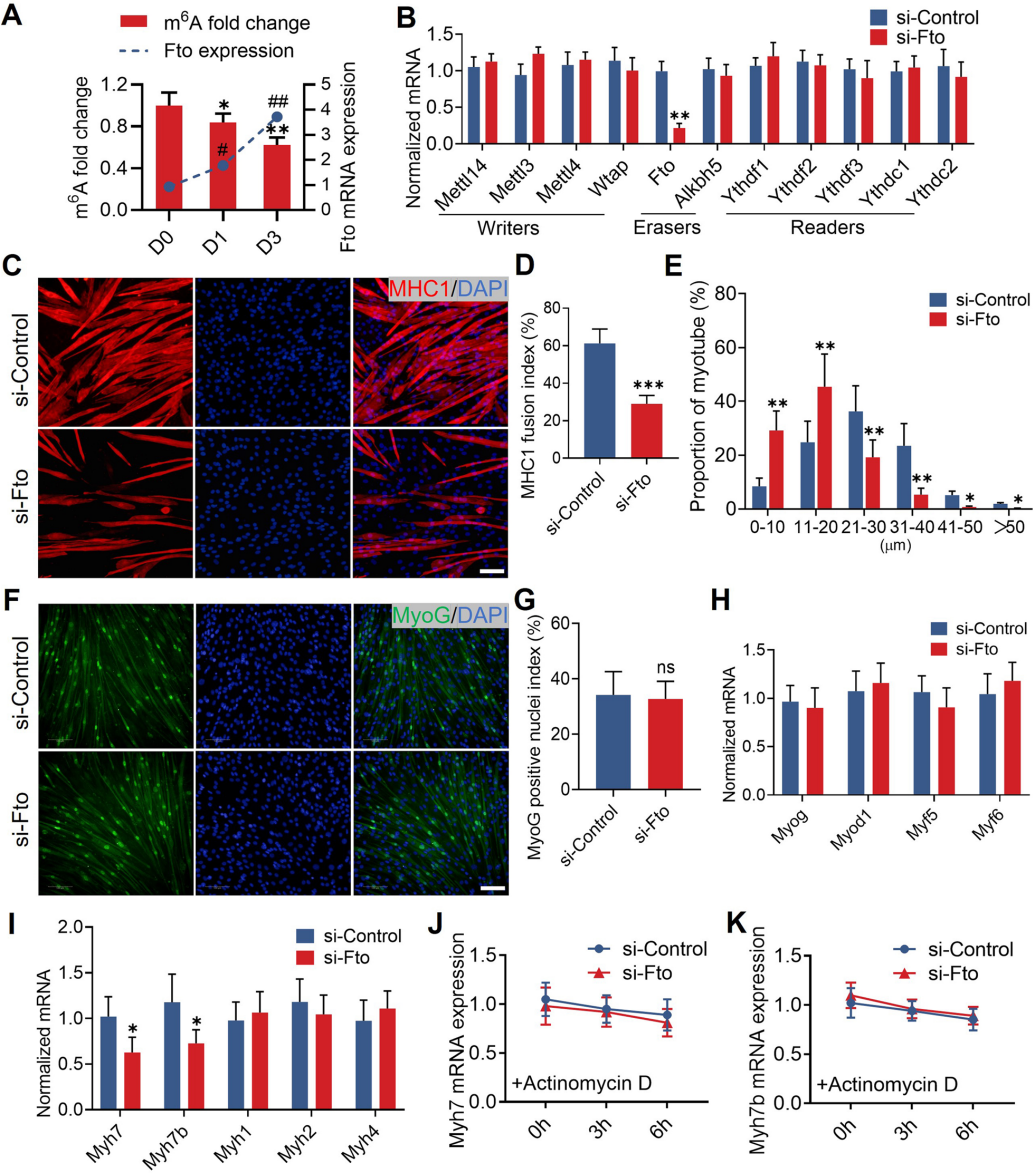
*Fto* maintains expression of murine slow-twitch fiber related genes in vitro. **A** During myogenic differentiation, the mRNA expression of *Fto* was gradually increased, and the m^6^A level gradually decreased. n = 5 in each case; data are represented as mean ± s.d. **B** Quantitative real-time PCR analysis showed that the expression of m^6^A-related genes was undisturbed after *Fto* knock-down. n = 5 in each case; data are represented as mean ± s.d. **C** Representative immunohistochemical images of MHC1 (red) during myogenic differentiation. Scale bar, 100 μm. **D** Immunofluorescence revealed that the MHC1 fusion index was significantly decreased in si-*Fto* treated myoblasts. n = 5 in each case; data are represented as mean ± s.d. **E** Immunofluorescence revealed that myotube width was significantly decreased in si-*Fto* treated myoblasts. n = 5 in each case; data are represented as mean ± s.d. **F** Representative immunohistochemical images of MyoG (green) during myogenic differentiation. Scale bar, 100 μm. **G** Immunofluorescence revealed no significant difference for the MyoG-positive nucleus index in si-*Fto* treated myoblasts. n = 5 in each case; data are represented as mean ± s.d. **H** Quantitative real-time PCR analysis showed that the expression of myogenic regulatory factors was undisturbed after Fto knock-down. n = 5 in each case; data are represented as mean ± s.d. **I** Quantitative real-time PCR analysis revealed that si-*Fto* interference restrained the expression of slow-twitch fiber related genes, including *Myh7* and *Myh7b*. n = 5 in each case; data are represented as mean ± s.d. **J** *Myh7* mRNA stability showed no difference in si-*Fto* treated myoblasts. n = 5 in each case; data are represented as mean ± s.d. **K** *Myh7b* mRNA stability showed no difference in si-*Fto* treated myoblasts. n = 5 in each case; data are represented as mean ± s.d. *P < 0.05, **P < 0.01, ***P < 0.001,^#^*P* < 0.05, ^##^*P* < 0.01

During our preliminary experiments, we found that the first three days of myogenic differentiation exhibited the most pronounced fluctuations in gene expression, particularly for *Fto* and various myogenic regulatory factors. Meanwhile, on days 4 and 5, as the myogenic differentiation program completed, these regulatory factors tended to stabilize (data not shown). Therefore, we chose to perform various assays on the third day of differentiation to investigate the beneficial function of *Fto* during myogenic differentiation. We quantified the m^6^A level and the expression of *Fto* during the myogenic differentiation process. We found gradually increased *Fto* expression but decreased m^6^A level (Fig. 2A), suggesting that *Fto* was a key regulator of m^6^A level. In addition, the expression of m^6^A-related genes was unchanged after the knockdown of *Fto* (Fig. 2B). Immunofluorescence revealed that the fusion index of MHC1 and myotube width were significantly decreased in si-*Fto* treated murine myoblasts (Fig. 2C–E). However, neither the proportion of MyoG-positive nuclei (Fig. 2F, G) nor the expression of myogenic regulatory factors (*Myog*, *Myod1*, *Myf5*, and *Myf6*) showed any discernible changes (Fig. 2H). It was interesting to note that si-*Fto* treatment reduced the expression of *Myh7* and *Myh7b*, which were primarily expressed in murine slow-twitch fibers (Fig. 2I). Additional experiments were conducted to test whether *Fto* affects the stability of RNA transcripts of *Myh7* and *Myh7b*, which did not show any significant differences in si-*Fto* treated murine myoblasts (Fig. 2J–K). These findings imply that *Fto* maintains the expression of genes relevant to murine slow-twitch fibers in an indirect way.

### *Fto* contributes to maintain the formation of murine slow-twitch fiber in vivo

**Fig. 3:**
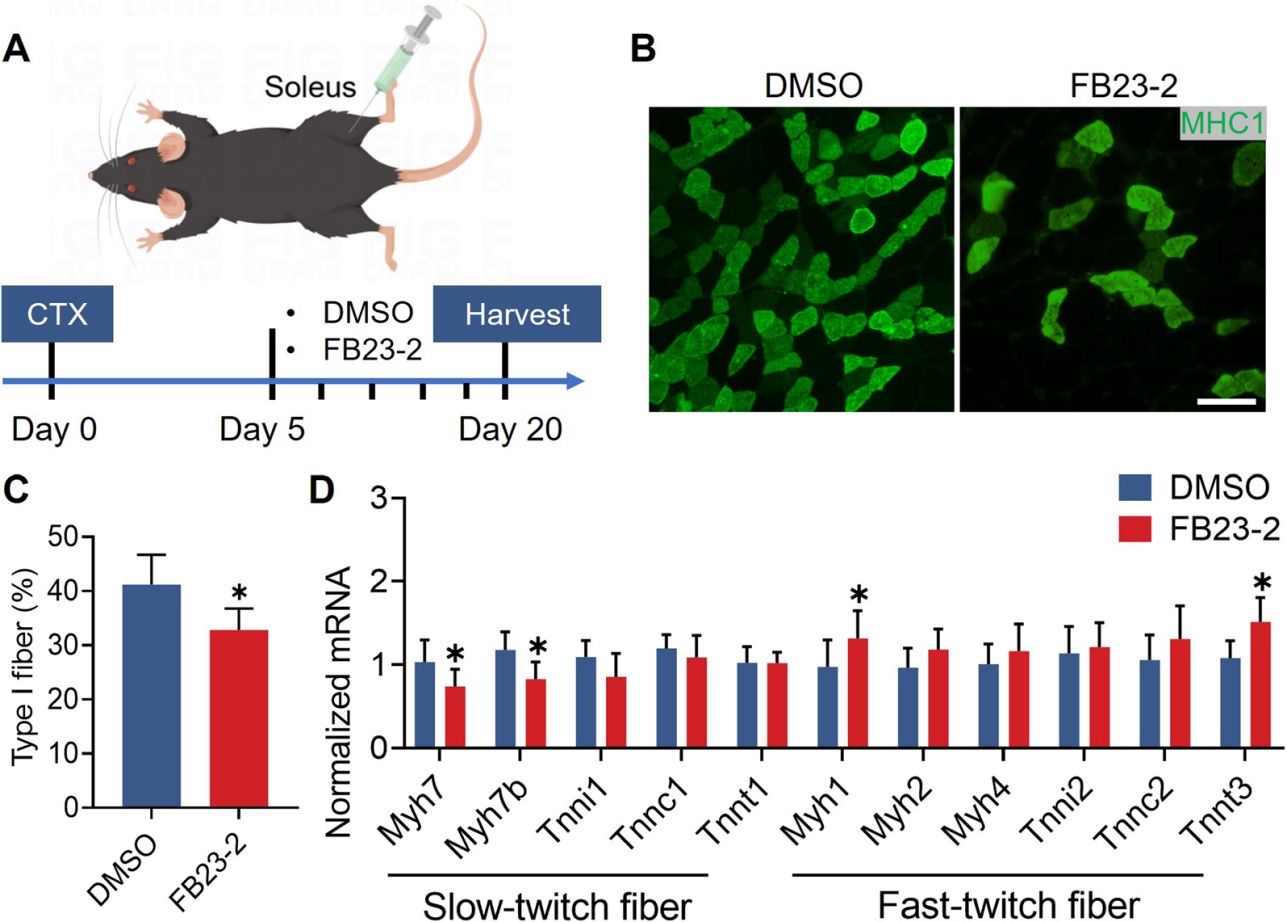
*Fto* maintains the formation of murine slow-twitch fiber in vivo. **A** Flow chart of the experiment on C57/BL mice. **B** Representative immunohistochemical images of MHC1 (green) in the soleus. Scale bar, 100 μm. **C** Immunofluorescence revealed that the percentage of type I muscle fiber was significantly decreased after treatment with FB23-2. n = 5 in each case; data are represented as mean ± s.d. **D** Quantitative real-time PCR analysis revealed that FB23-2 treatment decreased the mRNA expression of *Myh7* and *Myh7b* in the soleus. n = 5 in each case; data are represented as mean ± s.d. *P < 0.05

To address the potential function of *Fto* in murine muscle fiber remodeling in vivo, we induced acute muscle injury in mice. FB23-2, a selective inhibitor of the FTO m^6^A demethylase, was administered into the soleus every 3 days **(**Fig. 3A). Immunofluorescence revealed that the percentage of type I muscle fibers was significantly decreased in the FB23-2-treated soleus (Fig. 3B, C). In addition, we detected significantly decreased levels of *Myh7* and *Myh7b* but decreased levels of *Myh1* and *Tnnt3* expression in this soleus (Fig. 3D). Collectively, these results reveal that *Fto* contributes to maintain murine slow-twitch fiber formation in vivo.

### *Fto* regulates murine muscle fiber remodeling in an *Nfatc1*–*Ythdf2*-dependent manner

**Fig. 4:**
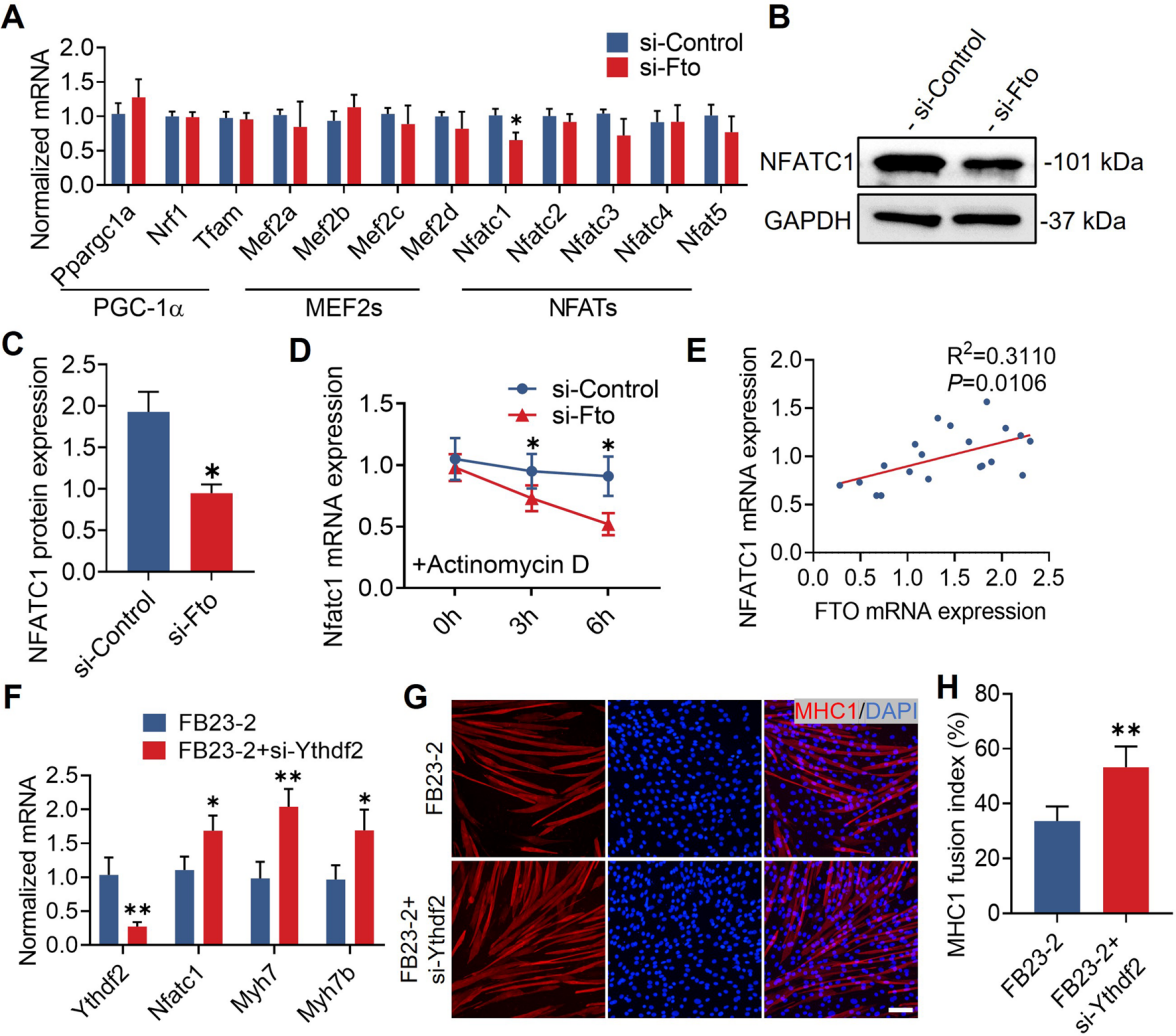
FTO regulates murine slow-twitch fiber related genes in a *NFATC1*–YTHDF2*-* dependent manner. **A** Quantitative real-time PCR analysis revealed that the mRNA expression of *Nfatc1* was significantly decreased in si-*Fto* treated myoblasts during myogenic differentiation. n = 5 in each case; data are represented as mean ± s.d. **B** Representative Western blot of NFATC1 in si-*Fto* treated myoblasts during myogenic differentiation. **C** Western blot quantification showed that NFATC1 was significantly decreased in si-*Fto* treated myoblasts. n = 3 in each case; data are represented as mean ± s.d. **D** The mRNA stability of *Nfatc1* was significantly decreased si-*Fto* treated myoblasts. n = 5 in each case; data are represented as mean ± s.d. **E** The mRNA expression of *FTO* was positively correlated with the expression level of *NFATC1* in the paraspinal muscle of adolescent idiopathic scoliosis. n = 20 in each case; data are represented as mean. **F** Quantitative real-time PCR analysis revealed that mRNA expression of *Ythdf2* was significantly decreased but *Nfatc1*, *Myh7*, and *Myh7b* were increased in si-*Ythdf2* silenced myoblasts with FB23-2 treatment. n = 5 in each case; data are represented as mean ± s.d. **G** Representative immunohistochemical images of MHC1 (red) in si-Ythdf2 silenced myoblasts with FB23-2 treated during myogenic differentiation. Scale bar, 100 μm. **H** Immunofluorescence revealed that the MHC1 fusion index was significantly increased in si-Ythdf2 silenced myoblasts with FB23-2 treated. n = 5 in each case; data are represented as mean ± s.d. *P < 0.05 and **P < 0.01

To identify the m^6^A demethylating targets of *Fto*, we examined the mRNA levels of several key regulators known to be involved in muscle fiber remodeling, including Pgc-1 (*Ppargc1a*, *Nrf1*, and *Tfam*), Mef2s (*Mef2a*, *Mef2b*, *Mef2c*, and *Med2d*), and Nfats (*Nfatc1*, *Nfatc2*, *Nfatc3*, *Nfatc4*, and *Nfat5*) [15, 16], and murine myoblasts treated with si-*Fto* substantially reduced the expression of *Nfatc1* (Fig. 4A). Recent research studies have indicated that nuclear accumulation of *Nfatc1* may trigger the activation of muscle remodeling genes. Moreover, *Nfatc1* governs the composition of fiber type and is necessary for the transformation of fast-to-slow fiber type in response to exercise in vivo [17, 18]. We confirmed the decreased level of NFATC1 with Western blot analysis (Fig. 4B, C), and the mRNA stability of *Nfatc1* in si-*Fto* treated myoblasts was significantly reduced (Fig. 4D). Additionally, we measured the level of *NFATC1* in the concave paravertebral muscles of 20 AIS patients, and we discovered a positive correlation between *FTO* and *NFATC1* (Fig. 4E). These findings demonstrate that *Fto* regulates the expression of *Nfatc1* in a demethylation-dependent way. YTHDF2 has been shown to destabilize mRNAs as the RNA m^6^A reader [19]. To investigated whether *Fto*-mediated *Nfatc1* mRNA degradation relied on m^6^A reader protein YTHDF2, we performed the rescue experiment by silencing endogenous *Ythdf2* during myogenic differentiation after FB23-2 was added. We found the expression of *Nfatc1*, *Myh7*, and *Myh7b* was restored after the treatment with si-*Ythdf2* (Fig. 4F). In addition, immunofluorescence showed that murine myoblasts treated with si-*Ythdf2* had a considerably increased MHC1 fusion index (Fig. 4G, H). Collectively, these data suggest that *Fto* regulates murine muscle fiber remodeling in an *Nfatc1*–*Ythdf2*-dependent manner.

### Asymmetric expression of FTO in AIS paravertebral muscles

**Fig. 5:**
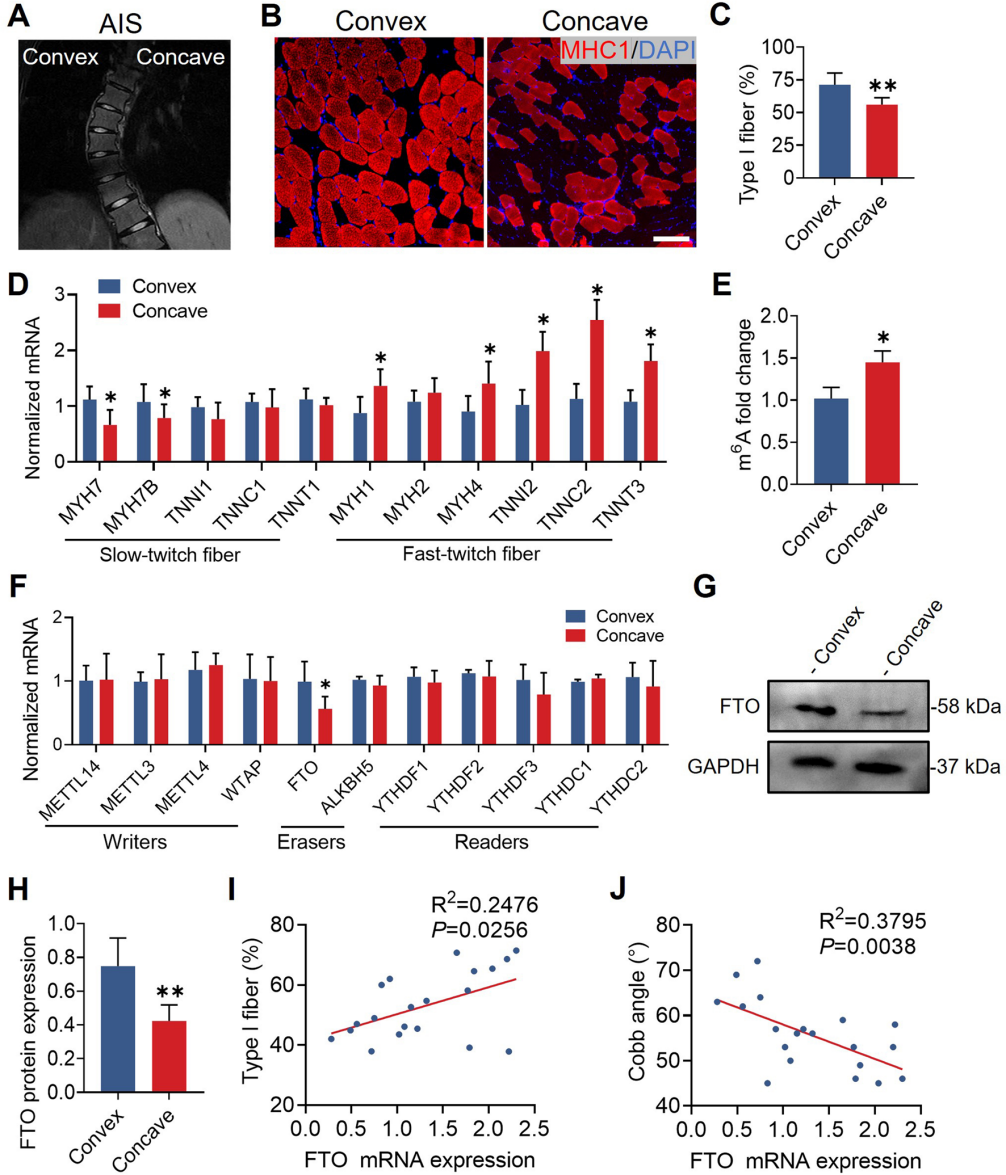
Reduced FTO in the concave paraspinal muscle of adolescent idiopathic scoliosis. **A** Magnetic resonance imaging showed convex and concave sides of adolescent idiopathic scoliosis. **B** Representative immunohistochemical images of MHC1 (red) in paraspinal muscle. Scale bar, 100 μm. **C** Immunofluorescence showed that the percentage of type I fibers was significantly reduced in concave paraspinal muscle. n = 5 in each case; data are represented as mean ± s.d. **D** Quantitative real-time PCR analysis showed that mRNA expression of *MYH7* and *MYH7B* was decreased, but *MYH1*, *MYH4*, *TNNI2*, *TNNC2* and *TNNT3* mRNA levels were increased in concave paraspinal muscle. n = 5 in each case; data are represented as mean ± s.d. **E** The m^6^A level was significantly increased in concave paraspinal muscle. n = 5 in each case; data are represented as mean ± s.d. **F** Quantitative real-time PCR analysis revealed that mRNA expression of m^6^A-related genes, and only *FTO* was significantly decreased in concave paraspinal muscle. n = 5 in each case; data are represented as mean ± s.d. **G** Representative Western blot of FTO in paraspinal muscle. **H** Western blot quantification showed that FTO was significantly decreased in concave paraspinal muscle. n = 5 in each case; data are represented as mean ± s.d. **I** The level of mRNA expression of *FTO* was positively correlated with the proportion of type I fiber. n = 20 in each case; data are represented as mean. **J** The mRNA expression level of *FTO* was negatively correlated with the Cobb angle of scoliosis. n = 20 in each case; data are represented as mean. *P < 0.05, **P < 0.01

We collected AIS paraspinal muscle biopsies from the concave and convex sides of the curve apex during surgery (Fig. 5A). Immunofluorescence revealed that the percentage of type I muscle fibers was significantly decreased on the concave side (Fig. 5B, C). In addition, quantitative real-time PCR analysis showed that the mRNA levels of *MYH7* and *MYHB*, which were predominantly expressed in slow-twitch fibers, were decreased on the concave side, while the mRNA levels of *MYH1*, *MYH4*, *TNNI2*, *TNNC2*, and *TNNT3*, which were predominantly expressed in fast-twitch fibers, were increased on the concave side (Fig. 5D). Collectively, our findings show that AIS paravertebral muscles have an asymmetric fiber type.

We attempted to further explain the fiber type discrepancy by evaluating the m^6^A methylation level in AIS paravertebral muscles, and we discovered that the concave side had a significantly higher level of m^6^A than the convex side (Fig. 5E). We further assessed the mRNA levels of many known regulators linked to m^6^A methylation writers (*METTL3*, *METTL4*, *METTL14*, and *WTAP*), erasers (*FTO* and *ALKBH5*), and readers (*YTHDF1*, *YTHDF2*, *YTHDF3*, *YTHDC1*, and *YTHDC2*). The expression of FTO was considerably downregulated in the concave side of AIS paravertebral muscles, according to quantitative real-time PCR and Western blot analysis (Fig. 5F–H). Additionally, we measured the mRNA levels of *FTO* in the concave paravertebral muscles of 20 AIS patients. We found a positive correlation between the mRNA level of *FTO* and the fraction of type I fibers, and a negative correlation between the mRNA level of *FTO* and the Cobb angle of scoliosis (Fig. 5I, J). These findings suggest that FTO might be involved in muscle fiber remodeling.

### Asymmetric expression of FTO in CS paravertebral muscles

**Fig. 6:**
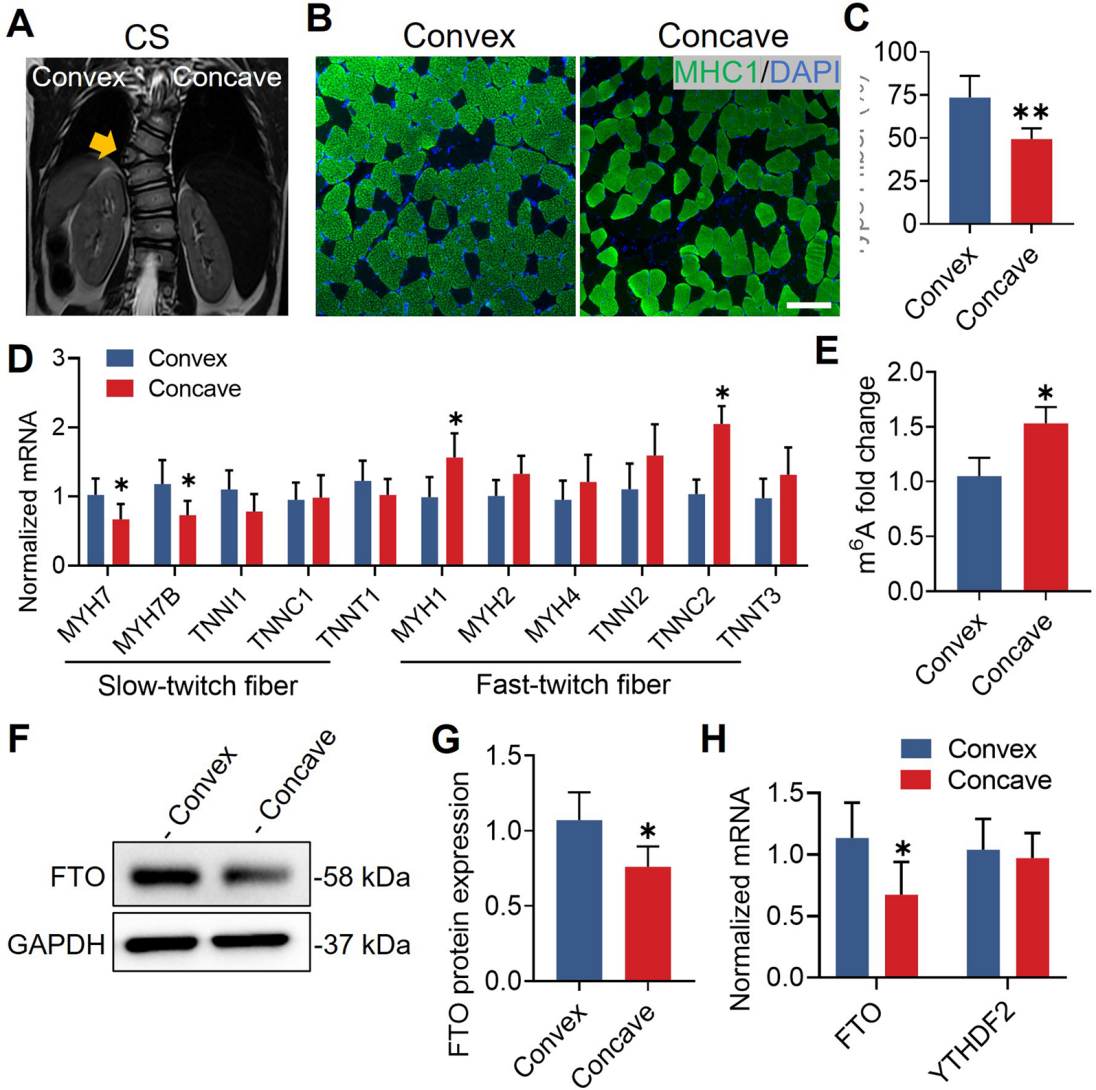
Reduced FTO in the concave paraspinal muscle of congenital scoliosis. **A** Magnetic resonance imaging showed convex and concave curves of congenital scoliosis, and the yellow arrow showed hemivertebra deformity. **B** Representative immunohistochemical images of MHC1 (green) in paraspinal muscle. Scale bar, 100 μm. **C** Immunofluorescence showed that the percentage of type I muscle fiber was significantly reduced in concave paraspinal muscle. n = 5 in each case; data are represented as mean ± s.d. **D** Quantitative real-time PCR analysis showed that mRNA expression of *MYH7* and *MYH7B* was decreased, but *MYH1* and *TNNC2* mRNA levels were increased in concave paraspinal muscle. n = 5 in each case; data are represented as mean ± s.d. **E** The m^6^A level was significantly increased in concave paraspinal muscle. n = 5 in each case; data are represented as mean ± s.d. **F** Representative Western blot of FTO in paraspinal muscle. **G** Western blot quantification showed that FTO was significantly decreased in concave paraspinal muscle. n = 5 in each case; data are represented as mean ± s.d. **H** Quantitative real-time PCR analysis showed that *FTO* mRNA expression was decreased in concave paraspinal muscle. n = 5 in each case; data are represented as mean ± s.d. *P < 0.05, **P < 0.01

In CS, a clear spinal deformity could be found (Fig. 6A), and the asymmetric vertebral body was the primary factor contributing to the development of scoliosis [20]. We, therefore, examined the paravertebral muscles in CS to ascertain whether the asymmetry of the muscle fibers was specific to AIS. Similar to AIS, we discovered that the concave side had a much lower percentage of type I muscle fibers (Fig. 6B, C). Additionally, quantitative real-time PCR analysis revealed that *MYH7* and *MYHB* expression levels were lower on the concave side, whereas *MYH1* and *TNNC2* expression levels were higher (Fig. 6D). We quantified the m^6^A methylation level in CS paravertebral muscles, and we found a considerably higher level of m^6^A on the concave side compared to the convex side (Fig. 6E). The expression of FTO was markedly downregulated in the concave side paravertebral muscles, according to quantitative real-time PCR and Western blot analysis (Fig. 6F–H). These data reveal that the asymmetric features of muscle fibers and FTO expression were consistent in AIS and CS.

### Physical exercise restores the asymmetric expression of FTO

**Fig. 7:**
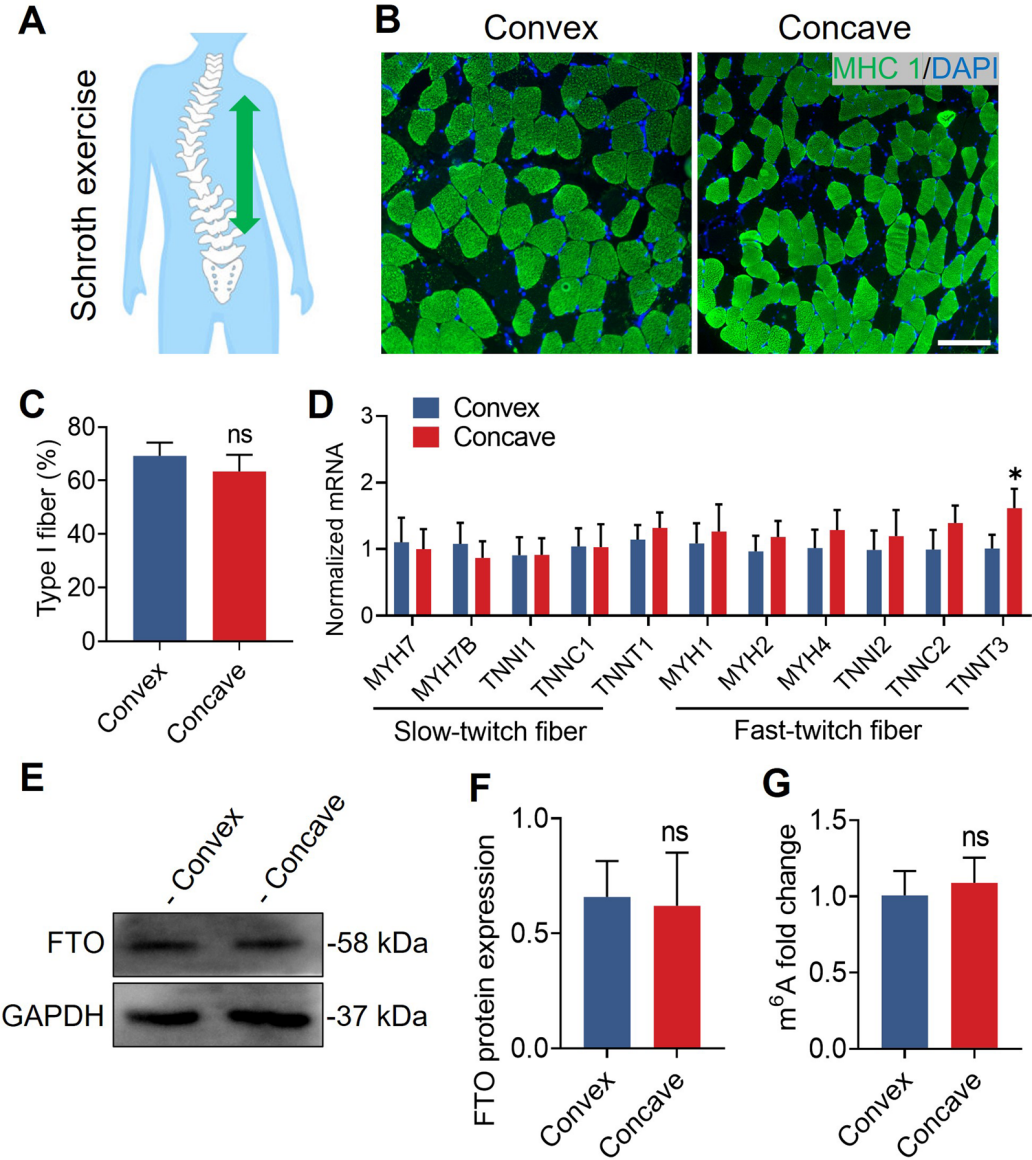
Physical exercise restores paraspinal muscle asymmetry. **A** Concave muscle activation was stretched after Schroth exercises. **B** Representative immunohistochemical images of type I muscle fiber (green) in paraspinal muscle from AIS patients who performed Schroth exercises longer than 3 months. **C** Immunofluorescence showed that the percentage of type I muscle fiber was comparable between convex and concave sides. n = 5 in each case; data are represented as mean ± s.d. Scale bar, 100 μm. **D** Quantitative real-time PCR analysis showed that *MYH7*, *MYHB*, *MYH1*, and *TNNI2*, which had an asymmetric expression in untrained AIS, were restored. n = 5 in each case; data are represented as mean ± s.d. **E** Representative Western blot for FTO expression between convex and concave sides. **F** Western blot quantification showed no significant difference in FTO expression between convex and concave sides. n = 5 in each case; data are represented as mean ± s.d. **G** The m^6^A level was comparable between convex and concave paraspinal muscle. n = 5 in each case; data are represented as mean ± s.d. *P < 0.05

Decreased electromyographic amplitudes have been found in the concave side paraspinal muscles of AIS [21, 22], and it is widely known that endurance training facilitates the change from fast to slow fiber types [23]. Since the Schroth method was first developed by Katharina Schroth in the 1920s, it has been widely used in scoliosis patients [24]. To investigate the beneficial effects of Schroth exercises on muscle fiber remodeling, we recruited five AIS patients who performed Schroth exercises for longer than 3 months (Fig. 7A). Immunofluorescence revealed that the percentage of type I muscle fibers was comparable between the convex and concave sides (Fig. 7B, C). In addition, quantitative real-time PCR analysis showed that the expression of *MYH7*, *MYH7B*, *MYH1*, and *TNNI2*, which were asymmetric in untrained AIS, was also restored (Fig. 7D). Interestingly, there was no difference in the m^6^A level or FTO expression (Fig. 7E–G). These data indicate that Schroth exercises partially restore the asymmetric expression of FTO and the percentage of type I muscle fibers. The reversible pattern of muscle fibers in AIS and the consistent paraspinal muscle features in AIS and CS suggest that the asymmetry of muscle fibers and FTO expression might not be the fundamental cause of AIS. Perhaps something intrinsic other than the susceptibility gene *FTO* is what causes AIS.

## Discussion

The asymmetry of paraspinal muscle in AIS has been demonstrated in several dimensions, including histology, electrophysiological activity, and magnetic resonance imaging. The asymmetric composition of muscle fibers is thought to be the driving force behind AIS. However, the present study suggests that *FTO* may not be the intrinsic factor driving the etiology of AIS. Instead, the related muscle fiber remodeling is more likely a secondary change after scoliosis. After comparing the paraspinal muscles of patients with AIS and CS, we observed a downregulation in the expression of FTO in the concave paraspinal muscles of both types of scoliosis. In AIS patients, we found a positive correlation between the mRNA level of *FTO* and the proportion of type I fibers, and Schroth exercises could restore the symmetrical muscle fiber ratio and FTO expression. Mechanistically, we provided experimental evidence from in vivo mouse models and in vitro mouse cell lines, demonstrating that FTO selectively interacts with NFATC1 to prevent its degradation and promote the expression of murine slow-type fiber genes in an NFATC1–YTHDF2-dependent manner.

Researchers have been baffled by the cause-and-effect link between AIS and the asymmetry of its paraspinal muscles for a long time [25]. In a previous study, a reduced proportion of type I muscle fibers was found on the concave side of AIS [22]. We also identified the downregulation of type I muscle fibers and mRNA expression of *MYH1*, *MYH4*, *TNNI2*, *TNNC2*, and *TNNT3* on the concave side of AIS. In addition, as a reference standard, we examined the paravertebral muscles in CS with a definite hemivertebral deformity. Surprisingly, the characteristics of paraspinal muscle asymmetry between these two types of scoliosis were highly consistent. Moreover, we investigated the paraspinal muscles of AIS patients who performed Schroth exercises, and the asymmetric muscle fibers were restored. Exercise enhances muscle performance and endurance by increasing the proportion of slow oxidative fibers, but muscle disuse causes type I fibers atrophy with a slow-to-fast fiber type shift [23, 26]. This reverse pattern suggests that the asymmetry of muscle fibers may not be the primary cause of AIS and that a possible explanation for the decreased proportion of slow-twitch fibers in concave paraspinal muscles is a secondary adaptation due to its chronic low-load demand.

The distinctive feature of AIS Is a low lean mass with no discernible vertebral deformities [27, 28]. The locus rs12149832 near *FTO* has been identified as significantly associated with AIS [8]. The risk allele of rs12149832 is associated with decreased expression of FTO [8, 27], and previous studies have shown that *FTO* is required for myogenesis [13, 14]. In this study, we investigated whether *FTO* was involved in the asymmetry of paraspinal muscle, which could indicate a potential role in the etiology of AIS. Consistent with the asymmetric pattern of muscle fiber proportions, the expression of FTO was significantly decreased on the concave side in AIS and CS, and Schroth exercises partially restored the asymmetric expression of FTO in AIS. These results suggest that perhaps something intrinsic other than the susceptibility gene *FTO* is what causes AIS.

Muscle fiber remodeling is a reversible process, and it depends on the exact coordination of metabolic and contractile gene expression programs to control fiber type specification [15]. Interestingly, we found that the mRNA level of *FTO* was positively correlated with the proportion of type I fibers in AIS. FTO has a reversible function in relevant RNA modifications [29], and its coordinated changes with muscle fiber asymmetry suggest that FTO may be involved in fiber type remodeling. In our study, the knockdown of *Fto* did not affect the mRNA expression of myogenic regulatory factors, such as *Myog*, *Myod1*, *Myf5*, and *Myf6*. It was noteworthy that slow-twitch fiber development was inhibited by *Fto* knockdown both in vitro and in vivo, and that *Myh7* and *Myh7b* expression—which was predominantly expressed in slow-twitch fibers—was regulated. These findings imply that FTO promotes the expression of genes relevant to murine slow-twitch fibers.

Muscle fiber remodeling has been studied in extensive detail in the past, and mitochondrial dysfunction could impair the function of skeletal muscle [30]. In addition, endurance training increases the intracellular calcium concentration, which activates calcineurin/NFATs and MEF2s. These two sets of transcription factors are primarily responsible for fiber remodeling and muscle formation [31]. In the present study, FTO promoted the expression of *Myh7* and *Myh7b*, but mRNA stability analysis indicated that they were not directly regulated by FTO demethylation. We further screened the core regulators of muscle fiber remodeling, including mitochondrial biogenesis, NFATs, and MEF2s [32], and *NFATC1* was identified as the m^6^A demethylating target of FTO during myogenic differentiation. NFATC1, which controls fiber type composition, is required for rapid to slow fiber type switching in response to exercise [18]. Moreover, YTHDF2 was identified as the m^6^A reader to degrade *NFATC1* mRNA. Mechanistically, we provided evidence from in vivo mouse models and in vitro mouse cell lines, showing that FTO participates in the murine muscle fiber remodeling process in an NFATC1–YTHDF2-dependent way.

It is essential to recognize its limitations. Firstly, due to the constraints in sample availability, we were unable to investigate the composition of paraspinal muscles in early AIS cases with a curvature range of 10°–20°. Secondly, we implemented a rigorous selection criterion and a paired methodology that inevitably led to a smaller sample size. Lastly, our mechanism study utilized a mouse tibialis anterior injury model and a mouse cell line rather than using myotubes sourced from patients with AIS. These limitations should be taken into consideration when interpreting our results.

## Conclusion

FTO supported the formation of murine slow-twitch fibers in an NFATC1–YTHDF2-dependent manner. The consistent paraspinal muscle features in AIS and CS and reversible pattern of muscle fibers in AIS suggest that the asymmetry of muscle fibers might not be the fundamental cause of AIS. Perhaps something intrinsic other than the susceptibility gene *FTO* is what causes AIS. Instead, *FTO* may contribute to the muscle fiber remodeling secondary to scoliosis.

## Supplementary Information

**Additional file 1**. **Table S1**: The patient characteristics of adolescent idiopathic scoliosis and congenital scoliosis. AIS, adolescent idiopathic scoliosis; CS, congenital scoliosis.

**Additional file 2**. **Table S2**: The target sequences of siRNAs for *Fto* and *Ythdf2*.

**Additional file 3**. **Table S3**: The real time quantitative PCR primers for human.

**Additional file 4**. **Table S4**: The real time quantitative PCR primers for mouse.

## Data Availability

The datasets used and/or analysed during the current study are available from the corresponding author on reasonable request.

## Abbreviations

AIS: Adolescent idiopathic scoliosis
CS: Congenital scoliosis
FTO: Fat mass and obesity-associated gene
CTX: Cardiotoxin
