# Supplementary material for: FTO-dependent m6A regulates muscle fiber remodeling in an NFATC1–YTHDF2 dependent manner
Source: Clin Epigenetics. 2023 Jul 5;15:109. doi: 10.1186/s13148-023-01526-5 (PMC10320966; doi:10.1186/s13148-023-01526-5)
Supplement: Supplementary file 1 — Additional file 1. Table S1: The patient characteristics of adolescent idiopathic scoliosis and congenital scoliosis. AIS, adolescent idiopathic scoliosis; CS, congenital scoliosis. [file 13148_2023_1526_MOESM1_ESM.docx]

| **Parameter** | **AIS without**  **Schroth exercises**  **(n=20)** | **CS**  **(n=5)** | **AIS with**  **Schroth exercises**  **(n=5)** |
| --- | --- | --- | --- |
| Lenke classification (1/2/3/4/5/6) | 12/4/2/2/0/0 | - | 3/2/0/0/0/0 |
| Sex (Female) | 20 | 5 | 5 |
| Cobb angle of major curve (°) | 53.2±4.6 | 46.8±7.9 | 47.4±3.9 |
| Age (Year) | 14.5±1.2 | 12.6±2.1 | 14.6±1.1 |

**Supplemental Table 1.** The patient characteristics of adolescent idiopathic scoliosis and congenital scoliosis. AIS, adolescent idiopathic scoliosis; CS, congenital scoliosis.
