# Supplementary material for: FTO-dependent m6A regulates muscle fiber remodeling in an NFATC1–YTHDF2 dependent manner
Source: Clin Epigenetics. 2023 Jul 5;15:109. doi: 10.1186/s13148-023-01526-5 (PMC10320966; doi:10.1186/s13148-023-01526-5)
Supplement: Supplementary file 2 — Additional file 2. Table S2: The target sequences of siRNAs for Fto and Ythdf2. [file 13148_2023_1526_MOESM2_ESM.docx]

| **Name** | **Target sequences** | |
| --- | --- | --- |
| *si-Fto#1* | *GGAACAAAGGAGTGAGATT* | |
| *si-Fto#2* | | *CAACAGGCACCTTGGATTA* |
| *si-Fto#3* | | *CATCGAGACACCAGGATTA* |
| *si-Ythdf2#1* | | *CTCCTACTTACCCAGTTACTACA* |
| *si-Ythdf2#2* | | *ATGATTTCGAACCTTACTTGAGC* |
| *si-Ythdf2#3* | | *CCGTTCCATTAAGTATAATATCT* |

**Supplemental Table 2.** The target sequences of siRNAs for *Fto* and *Ythdf2.*
