# Supplementary material for: FTO-dependent m6A regulates muscle fiber remodeling in an NFATC1–YTHDF2 dependent manner
Source: Clin Epigenetics. 2023 Jul 5;15:109. doi: 10.1186/s13148-023-01526-5 (PMC10320966; doi:10.1186/s13148-023-01526-5)
Supplement: Supplementary file 3 — Additional file 3. Table S3: The real time quantitative PCR primers for human. [file 13148_2023_1526_MOESM3_ESM.docx]

| **Gene** | **Primer** | |
| --- | --- | --- |
| *GAPDH, F* | *GGAGCGAGATCCCTCCAAAAT* | |
| *GAPDH, R* | *GGCTGTTGTCATACTTCTCATGG* | |
| *MYH1, F* | | *CCCTACAAGTGGTTGCCAGTG* |
| *MYH1, R* | | *CTTCCCTGCGCCAGATTCTC* |
| *MYH2, F* | | *AGAAACTTCGCATGGACCTAGA* |
| *MYH2, R* | | *CCAAGTGCCTGTTCATCTTCA* |
| *MYH4, F* | | *ACAAGGTTCTAAATGCGAGTGC* |
| *MYH4, R* | | *TGACCGAATTTGTACTGGGTG* |
| *MYH7, F* | | *CTTTGCTGTTATTGCAGCCATT* |
| *MYH7, R* | | *AGATGCCAACTTTCCTGTTGC* |
| *MYH7B, F* | | *TCGCTTCGACTTACTGGAGGA* |
| *MYH7B, R* | | *GGAGGCCGTATAGACTGGGA* |
| *TNNI1, F* | | *TCCGTGGGAAGTTCAAGCG* |
| *TNNI1, R* | | *GACTTGGCGGCATCAAACATC* |
| *TNNI2, F* | | *ATCTGCGGGGCAAGTTCAAG* |
| *TNNI2, R* | | *AGGACTCGGACTCAAACATCT* |
| *TNNC1, F* | | *TGGTTCGGTGCATGAAGGAC* |
| *TNNC1, R* | | *GTCGATGTAGCCATCAGCATT* |
| *TNNC2, F* | | *AGGAATGCAGACGGCTACATC* |
| *TNNC2, R* | | *AACTCGTCGAAGTCAATGCGG* |
| *TNNT1, F* | | *CAGAGGATGATGCCAAGAAAA* |
| *TNNT1, R* | | *TTACCACGCTTCTGTTCTGC* |
| *TNNT3, F* | | *AGGAGCTGGTCGCTCTCAA* |
| *TNNT3, R* | | *CCTTCTCTGCACGAATCCTCT* |
| *METTL14, F* | | *GAGTGTGTTTACGAAAATGGGGT* |
| *METTL14, R* | | *CCGTCTGTGCTACGCTTCA* |
| *METTL3, F* | | *AGATGGGGTAGAAAGCCTCCT* |
| *METTL3, R* | | *TGGTCAGCATAGGTTACAAGAGT* |
| *METTL4, F* | | *TTGTCACCCCTGCAAATACAG* |
| *METTL4, R* | | *TCCACAGACCAAGAGGGATAAAG* |
| *WTAP, F* | | *TTGTAATGCGACTAGCAACCAA* |
| *WTAP, R* | | *GCTGGGTCTACCATTGTTGATCT* |
| *FTO, F* | | *AACACCAGGCTCTTTACGGTC* |
| *FTO, R* | | *TGTCCGTTGTAGGATGAACCC* |
| *ALKBH5, F* | | *ATGCACCCCGGTTGGAAAC* |
| *ALKBH5, R* | | *GACTTGCGCCAGTAGTTCTCA* |
| *YTHDF1, F* | | *GGGGACAAGTGGGTCTCAAG* |
| *YTHDF1, R* | | *AGGGTGTCGCTGTGAAAGC* |
| *YTHDF2, F* | | *AGCCCCACTTCCTACCAGATG* |
| *YTHDF2 R* | | *TGAGAACTGTTATTTCCCCATGC* |
| *YTHDF3, F* | | *TCAGAGTAACAGCTATCCACCA* |
| *YTHDF3 R* | | *GGTTGTCAGATATGGCATAGGCT* |
| *YTHDC1, F* | | *AACTGGTTTCTAAGCCACTGAGC* |
| *YTHDC1 R* | | *GGAGGCACTACTTGATAGACGA* |
| *YTHDC2, F* | | *CAAAACATGCTGTTAGGAGCCT* |
| *YTHDC2 R* | | *CCACTTGTCTTGCTCATTTCCC* |
| *NFATC1, F* | | *TGTGCCGGAATCCTGAAACTC* |
| *NFATC1, R* | | *GAGCATTCGATGGGGTTGGAG* |

**Supplemental Table 3.** The real time quantitative PCR primers for human.
