# Supplementary material for: FTO-dependent m6A regulates muscle fiber remodeling in an NFATC1–YTHDF2 dependent manner
Source: Clin Epigenetics. 2023 Jul 5;15:109. doi: 10.1186/s13148-023-01526-5 (PMC10320966; doi:10.1186/s13148-023-01526-5)
Supplement: Supplementary file 4 — Additional file 4. Table S4: The real time quantitative PCR primers for mouse. [file 13148_2023_1526_MOESM4_ESM.docx]

| **Gene** | **Primer** |
| --- | --- |
| *Gapdh, F* | *AGGTCGGTGTGAACGGATTTG* |
| *Gapdh, R* | *TGTAGACCATGTAGTTGAGGTCA* |
| *Myh1, F* | *GGCAGCAGCAGCTGCGGAAGCAGAGTCTGG* |
| *Myh1, R* | *GAGTGCTCCTCAGATTGGTCATTAGC* |
| *Myh2, F* | *GGCACAAACTGCTGAAGCAGAGGC* |
| *Myh2, R* | *GCACCCATCCTCATTTCGTGA* |
| *Myh4, F* | *GGAATGGCACTTGCGTTTAACA* |
| *Myh4, R* | *CTGGACGATGTCTTCCATCTCTCC* |
| *Myh7, F* | *GCCAACTATGCTGGAGCTGATGCCC* |
| *Myh7, R* | *GGTGCGTGGAGCGCAAGTTTGTCATAAG* |
| *Myh7b, F* | *CATGGGATGGTAAGAAACGGG* |
| *Myh7b, R* | *TCCTCCAGTAAGTCGAAACGG* |
| *Tnni1, F* | *TGAAGCCAAATGCCTCCACAACAC* |
| *Tnni1, R* | *ACACCTTGTGCTTAGAGCCCAGTA* |
| *Tnni2, F* | *AGCAGCAAGGAGCTGGAAGA* |
| *Tnni2, R* | *ATGGCGTCGGCAGACATAC* |
| *Tnnc1, F* | *AGCTCATGAAGGACGGTGACAAGA* |
| *Tnnc1, R* | *AACCGTGCAAGACCAGCATCTACT* |
| *Tnnc2, F* | *CCATCATCGAGGAGGTGGAC* |
| *Tnnc2, R* | *CTTCCCCTTCGCATCCTCTT* |
| *Tnnt1, F* | *TGGATCCACCAGCTGGAATCAGAA* |
| *Tnnt1, R* | *GCTGATGCGGTTGTAGAGCACATT* |
| *Tnnt3, F* | *AACTGGAGACTGACAAATTCGAGT* |
| *Tnnt3, R* | *GCTGTGCTTCTGGGTTTGGT* |
| *Ppargc1a, F* | *AAGTGGTGTAGCGACCAATCG* |
| *Ppargc1a, R* | *AATGAGGGCAATCCGTCTTCA* |
| *Nfatc1, F* | *GACCCGGAGTTCGACTTCG* |
| *Nfatc1, R* | *TGACACTAGGGGACACATAACTG* |
| *Mettl14, F* | *CTGAGAGTGCGGATAGCATTG* |
| *Mettl14, R* | *GAGCAGATGTATCATAGGAAGCC* |
| *Mettl3, F* | *CTGGGCACTTGGATTTAAGGAA* |
| *Mettl3, R* | *TGAGAGGTGGTGTAGCAACTT* |
| *Mettl4, F* | *TGGGTGGTTACTGGATCATCT* |
| *Mettl4, R* | *AGGAGCAAAGCACATAGCAGG* |
| *Wtap, F* | *TAGACCCAGCGATCAACTTGT* |
| *Wtap, R* | *CCTGTTTGGCTATCAGGCGTA* |
| *Fto, F* | *TCATCATCCTTAACCACATGGTG* |
| *Fto, R* | *TGAAGACGATAGCTGTCATCCA* |
| *Alkbh5, F* | *CGCGGTCATCAACGACTACC* |
| *Alkbh5, R* | *ATGGGCTTGAACTGGAACTTG* |
| *Ythdf1, F* | *ACAGTTACCCCTCGATGAGTG* |
| *Ythdf1, R* | *GGTAGTGAGATACGGGATGGGA* |
| *Ythdf2, F* | *GAGCAGAGACCAAAAGGTCAAG* |
| *Ythdf2, R* | *CTGTGGGCTCAAGTAAGGTTC* |
| *Ythdf3, F* | *GATCAGCCTATGCCATATCTGAC* |
| *Ythdf3, R* | *CCCCTGGTTGACTAAAAACACC* |
| *Ythdc1, F* | *GTCCACATTGCCTGTAAATGAGA* |
| *Ythdc1, R* | *GGAAGCACCCAGTGTATAGGA* |
| *Ythdc2, F* | *ACCGACTAAGTCAATCTCTTGGT* |
| *Ythdc2, R* | *AGGCTCCTAACAGCATGTTTTG* |
| *Myog, F* | *GAGACATCCCCCTATTTCTACCA* |
| *Myog, R* | *GCTCAGTCCGCTCATAGCC* |
| *Myod1, F* | *CCACTCCGGGACATAGACTTG* |
| *Myod1, R* | *AAAAGCGCAGGTCTGGTGAG* |
| *Myf5, F* | *AAGGCTCCTGTATCCCCTCAC* |
| *Myf5, R* | *TGACCTTCTTCAGGCGTCTAC* |
| *Myf6, F* | *AGAGGGCTCTCCTTTGTATCC* |
| *Myf6, R* | *CTGCTTTCCGACGATCTGTGG* |
| *Mef2a, F* | *CAGGTGGTGGCAGTCTTGG* |
| *Mef2a, R* | *TGCTTATCCTTTGGGCATTCAA* |
| *Mef2c, F* | *ATCCCGATGCAGACGATTCAG* |
| *Mef2c, R* | *AACAGCACACAATCTTTGCCT* |
| *Mef2d, F* | *CGAGATCGCGCTCATCATCTT* |
| *Mef2d, R* | *AGCCGTTGAAACCCTTCTTCC* |
| *Ppargc1a, F* | *AAGTGGTGTAGCGACCAATCG* |
| *Ppargc1a, R* | *AATGAGGGCAATCCGTCTTCA* |
| *Nrf1, F* | *TATGGCGGAAGTAATGAAAGACG* |
| *Nrf1, R* | *TATGGCGGAAGTAATGAAAGACG* |
| *Tfam, F* | *ATTCCGAAGTGTTTTTCCAGCA* |
| *Tfam, R* | *TCTGAAAGTTTTGCATCTGGGT* |
| *Nfatc1, F* | *GACCCGGAGTTCGACTTCG* |
| *Nfatc1, R* | *TGACACTAGGGGACACATAACTG* |
| *Nfatc2, F* | *TCATCCAACAACAGACTGCCC* |
| *Nfatc2, R* | *GGGAGGGAGGTCCTGAAAACT* |
| *Nfatc3 F* | *CACCACTTTGCTTACCACATCA* |
| *Nfatc3, R* | *CACCACTTTGCTTACCACATCA* |
| *Nfatc4, F* | *GAGCTGGAATTTAAGCTGGTGT* |
| *Nfatc4, R* | *CATGGAGGGGTATCCTCTGAG* |
| *Nfat5, F* | *CAGCGCCCAATAGTTGGCA* |
| *Nfat5, R* | *TGCTGGTGAAAAATTGACTGGT* |

**Supplemental Table 4.** The real time quantitative PCR primers for mouse.
